# Evaluating Variations in Spinopelvic Parameters from Sitting to Standing: A Comparative Analysis of 1447 Older Adults Across Age, BMI, and Gender Subgroups

**DOI:** 10.3390/jcm14092952

**Published:** 2025-04-24

**Authors:** Atahan Durbas, Tejas Subramanian, Chad Simon, Myles R. J. Allen, Justin Samuel, Luis Felipe Colón, Michael R. Mazzucco, Cale Pagan, Theofilos Karasavvidis, Jonathan Vigdorchik, Matthew E. Cunningham, Han Jo Kim, Francis C. Lovecchio

**Affiliations:** 1 Department of Spine Surgery, Hospital for Special Surgery, 535 E 70th St, New York, NY 10021, USAlovecchiof@hss.edu (F.C.L.); 2 Weill Cornell Medical College, 1300 York Ave, New York, NY 10021, USA; 3 Department of Hip and Knee Replacement Surgery, Hospital for Special Surgery, 535 E 71st St, New York, NY 10021, USA

**Keywords:** sagittal alignment, sagittal balance, sitting, standing, sitting radiographs, spinopelvic, spinal deformity, pelvic incidence, lumbar lordosis, sacroiliac joint

## Abstract

**Background/Objectives**: Sagittal spinal alignment goals for adult spinal deformity (ASD) surgery are predominantly derived from standing radiographs, despite the biomechanical relevance of sitting posture. Existing studies on sitting alignment involve young, healthy cohorts, which poorly represent ASD patients. This study assessed posture-dependent changes in spinopelvic parameters, including pelvic incidence (PI), pelvic tilt (PT), sacral slope (SS), and lumbar lordosis (LL), and examined how age, BMI, and gender influence these changes. **Methods:** In this retrospective cohort study, sitting and standing lateral radiographs of 1447 patients were evaluated. Spinopelvic parameters were measured, and changes (ΔPI, ΔPT, ΔSS, and ΔLL) were calculated. Multiple regression analysis was used to determine associations between these changes and age, BMI, and gender. **Results**: All parameters differed significantly between positions (*p* < 0.001); PT and PI increased in sitting (ΔPT = −19.20°; ΔPI = −4.52°), while SS and LL increased in standing (ΔSS = 14.67°; ΔLL = 18.44°). Older age correlated with increased ΔPT (*p* < 0.001) and ΔPI (*p* = 0.049) but reduced ΔLL and ΔSS (*p* < 0.001). Higher BMI was associated with decreased ΔPI, ΔPT, and ΔLL (*p* < 0.001, 0.003, and 0.025). Females showed greater ΔPT (*p* = 0.013) but smaller ΔPI, ΔSS, and ΔLL (*p* = 0.043, <0.001, and 0.001). **Conclusions**: Spinopelvic parameters vary significantly between sitting and standing positions, affected by age, BMI, and gender. The observed PI change suggests SIJ involvement, highlighting the need for posture-specific and demographic-adjusted alignment goals in ASD surgery to optimize outcomes.

## 1. Introduction

Achieving optimal sagittal spinal alignment is a key goal in spinal fusion surgeries, as it has been consistently associated with better functional outcomes and quality of life improvements [1,2]. Current standards for determining ideal sagittal alignment are mainly based on radiographic measurements obtained from the standing position [3,4]. However, most recent studies demonstrated that sitting posture—adopted for 6–8 h daily in modern industrialized societies [5,6]—induces significant spinopelvic changes [7,8,9,10]. Transitioning from standing to sitting generates forces that cause the pelvis to retrovert around the hip joints and reduces lumbar lordosis, which then leads to compensatory changes to maintain an upright posture [10,11,12,13,14]. These dynamics are particularly relevant in the patient population of adult spinal deformity (ASD) surgery, who often exhibit limited compensatory capacity due to age-related stiffness or comorbid hip/knee osteoarthritis (OA) [15,16,17].

Previous studies showing sitting–standing changes in lumbar alignment and pelvic compensation primarily involved small samples of young, healthy cohorts [9,10,18], a demographic rarely encountered in ASD patients undergoing spinal reconstruction. This gap is concerning, as OA prevalence increases with age and BMI [19,20,21], potentially altering pelvic compensation and lumbar adaptability. Failure to account for these factors risks overcorrection during fusion—it may lead to suboptimal surgical outcomes [7,22,23] and potentially contribute to the rising incidence of surgical complications by exacerbating stress on adjacent segments [24,25].

This study aimed to identify differences in four spinopelvic parameters—pelvic incidence (PI), pelvic tilt (PT), sacral slope (SS), and lumbar lordosis (LL)—between sitting and standing positions. This study also evaluated how age, body mass index (BMI), and gender influence the extent of changes in these parameters. We hypothesized that older age, a higher BMI, and being male would correlate with decreased pelvic compensation and lumbar change capacity. By including individuals of both genders with a broad age range and varying BMI levels, this study sought to provide a more comprehensive understanding of posture-related changes in sagittal spinal alignment, which could help optimize surgical planning and improve postoperative outcomes in ASD.

## 2. Materials and Methods

### 2.1. Study Design and Patient Cohort

This study received approval from the Institutional Review Board (IRB) of the Hospital for Special Surgery (HSS). This retrospective cohort study, conducted at a single institution, reviewed data from 1613 adult patients (age ≥ 18 years) under consideration for hip replacement (HR) surgery between 2014 and 2021. Inclusion criteria required that each patient had both sitting and standing lateral lumbar preoperatively radiographs available, a coronal maximum Cobb angle < 20°, and the absence of spondylolisthesis or previous spinal fusion surgery history. The severity of hip osteoarthritis (HOA) for each patient was assessed using the Kellgren–Lawrence grading system [26]. According to this criterion, patients with Grade 4 OA were excluded. All included patients had at least 90 degrees of hip flexion, which was a clinical prerequisite for obtaining a sitting radiograph.

### 2.2. Data Collection

**Figure 1:**
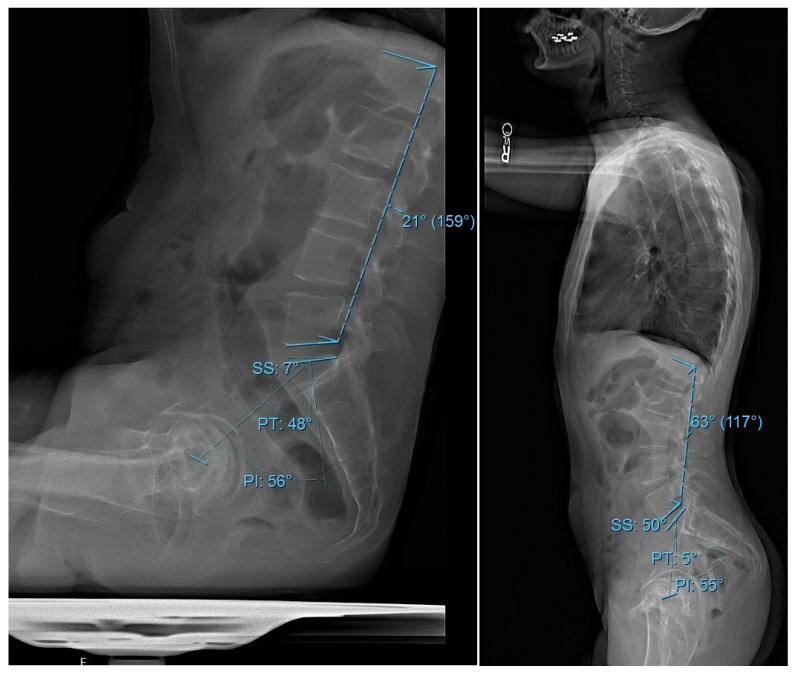
**Spinopelvic measurements in sitting and standing XRs.** Lateral radiographs of an idealized patient demonstrating the measurements of spinopelvic parameters. The parameters include pelvic incidence (PI), pelvic tilt (PT), sacral slope (SS), and lumbar lordosis (LL): on the left panel in sitting and on the right panel in standing.

Demographic and perioperative data were collected and managed using Research Electronic Data Capture (REDCap), hosted by the Weill Cornell Medicine Clinical and Translational Science Center (New York, NY, USA) and supported by the National Center for Advancing Translational Science (NCATS) of the National Institute of Health (NIH), (Bethesda, MD, USA). The analyzed variables included demographic factors such as patient age, gender, BMI, comorbidities, and the age-adjusted Charlson Comorbidity Index (CCI). Surgical and postoperative variables encompassed the American Society of Anesthesiologists (ASA) score and date of surgery. All radiographic measurements were performed using the institutional Picture Archiving and Communication System (PACS) software (Sectra AB, Linköping, Sweden; v12.3.7). Three independent, fellowship-trained spine surgeons conducted manual measurements using standardized PACS angle and line tools. If two observers differed in measurements by more than ±5°, the radiograph was jointly reviewed and a consensus measurement was recorded for consistency and accuracy. Preoperative radiographic parameters were PI, PT, SS, LL, and PI-LL in both sitting and standing positions (Figure 1).

### 2.3. Primary Outcome

Spinopelvic parameters, including PI, PT, SS, LL, and PI-LL, were measured for each image using both sitting and standing radiographs. All measurements were initially performed by a single observer and subsequently reviewed by a second observer to minimize interobserver variability. The primary outcome of this study was changes in spinopelvic parameters, changes shown as ΔPI, ΔPT, ΔSS, and ΔLL, between sitting and standing positions. These changes were calculated by subtracting the sitting values from the corresponding standing values for each parameter. Positive values indicated an increase in the parameter when transitioning from sitting to standing, while negative values indicated a decrease.

### 2.4. Statistical Analysis

The data were analyzed using R software (version 4.4.1; R Foundation for Statistical Computing, Vienna, Austria) with the *gtsummary* and *tidyverse* packages, with the statistical significance level set at *p* < 0.05. Descriptive statistics summarized the demographic and radiographic measurements, presenting continuous variables as medians with interquartile ranges (IQRs) or means with standard deviations (SDs) and categorical variables as frequencies and percentages. Comparisons between groups were performed using the Wilcoxon rank-sum test or paired t-test for continuous variables and Pearson’s chi-squared test or Fisher’s exact test for categorical variables, depending on the normality of variables calculated by the Shapiro–Wilk test. Subgroups were compared based on the analysis of variance (ANOVA) or Kruskal–Wallis, depending on the normality test. Multiple linear regression analyses were performed to determine the influence of age, BMI, and gender on these changes.

## 3. Results

### 3.1. Demographic Characteristics

The final study group included 1447 patients, consisting of 795 females (54.9%) and 652 males (45.1%). The mean age of the participants was 63.59 (±10.85) years, with a mean BMI of 28.81 (±5.70) kg/m^2^ and a mean PI of 51.67° (±10.75). A summary of sitting and standing spinopelvic measurements, along with the corresponding positional changes, is provided in Table 1.

**Figure 2:**
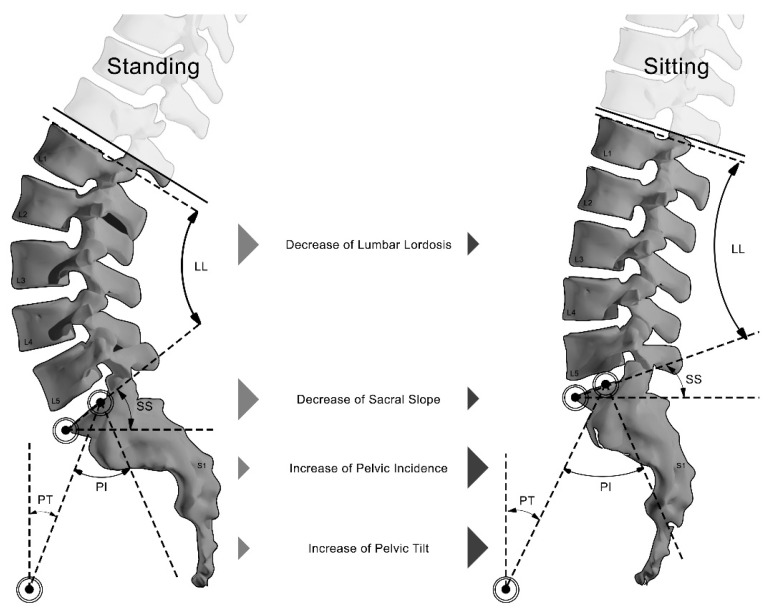
**Differences in spinal alignment between standing and sitting positions.** Schematizing the differences in the lumbar spine between sitting and standing positions. Arrows depict the direction and magnitude of the decrease in lumbar lordosis (LL) and sacral slope (SS) and the increase in pelvic incidence (PI) and pelvic tilt (PT).

PT and PI were higher in the sitting position, while SS and LL were higher in the standing position (Figure 2).

### 3.2. Subgroup Analysis

#### 3.2.1. Stratification by Age

Participants were divided into three age subgroups: under 60 years old (*n* = 490), 60–69 years old (*n* = 531), and 70 years or older (*n* = 426) (Table 2). While BMI was different between age subgroups (*p* < 0.001), gender distribution was similar (*p* = 0.05). Although PI values in sitting and standing positions were comparable among different subgroups (*p* = 0.20 and *p* = 0.74, respectively), ΔPI was different (*p* = 0.03). All other changes in spinopelvic parameters also differed (*p* < 0.001).

#### 3.2.2. Stratification by BMI

Participants were categorized into three BMI subgroups: normal weight (BMI < 25) (*n* = 397), overweight (25 ≤ BMI < 30) (*n* = 498), and obese (BMI ≥ 30) (*n* = 546). There was a difference in age and gender across the subgroups (*p* < 0.001 for both). Unlike the standing position, all sitting parameters differed among subgroups (Table 3). Also, except for ΔLL (*p* = 0.07), ΔPI, ΔPT, and ΔSS were different between subgroups (*p* < 0.001, *p* < 0.001, and *p* = 0.003, respectively).

#### 3.2.3. Stratification by Gender

Females were older than males on average (*p* = 0.01), and males had a higher mean BMI compared to females (*p* < 0.001) (Table 4). In the standing position, PT and LL values differed between genders (*p* < 0.001 and *p* = 0.002). However, in the sitting position, differences were observed between the two genders in SS, LL, and PI-LL values (*p* = 0.04, *p* < 0.001, and *p* = 0.02, respectively). Except for ΔPI, all changes were different between gender subgroups (all *p* < 0.001).

### 3.3. Multiple Regression Analysis

Four separate multiple linear regression models were conducted to assess the impact of age, BMI, and gender on changes in spinopelvic parameters. Age was independently associated with all four changes. Increasing age was associated with increased ΔPI (*p* = 0.049) and ΔPT (*p* < 0.001). Conversely, it was linked with decreased ΔLL and ΔSS (*p* < 0.001 for both). Higher BMI correlated with decreased ΔPI, ΔPT, and ΔLL (*p* < 0.001, *p* = 0.003, and *p* = 0.03, respectively). In addition, females were associated with greater ΔPT (*p* = 0.01), yet smaller ΔPI, ΔSS, and ΔLL (*p* = 0.04, *p* < 0.001, and *p* = 0.001, respectively).

## 4. Discussion

Despite the growing recognition of posture-dependent spinopelvic dynamics, alignment guidelines and surgical planning often rely on measurements obtained in the standing position. This can neglect significant postural changes that occur with sitting, a common position for elderly or mobility-impaired postoperative patients. Previous studies on spinal alignment in sitting versus standing primarily involved young, healthy individuals, limiting their relevance to older or reduced-mobility populations. For instance, studies on healthy cohorts consistently demonstrate reduced lumbar lordosis (LL), decreased sacral slope (SS), and increased pelvic tilt (PT) in sitting compared to standing, highlighting the dynamic nature of spinopelvic alignment [27,28]. However, these studies generally lack the representation of older adults, such as those undergoing ASD surgery. Our study aims to evaluate sitting–standing differences in key spinopelvic parameters across a diverse patient group to better represent ASD patients and to assess how demographic factors like age, BMI, and gender influence these changes.

This study expands on the literature by evaluating posture-dependent changes in spinopelvic parameters (PI, PT, SS, and LL) across 1447 patients undergoing HR surgery, a population with more similar demographic and functional profiles to ASD patients. Our findings revealed (1) dynamic changes in PI between sitting and standing, challenging its classification as a fixed parameter; (2) age-related reductions in lumbar adaptability; (3) obesity-associated restrictions in pelvic motion; and (4) gender-specific differences in postural compensation. These findings highlight the importance of demographic factors in spinopelvic alignment and their implications for optimal surgical planning.

This study demonstrated significant changes in PT, SS, and LL from standing to sitting position, consistent with previous studies [7,8,9,10], which have consistently shown that transitioning from standing to sitting results in a substantial reduction in LL, along with a marked increase in PT. For instance, the radiographic study of 30 healthy male volunteers demonstrated that LL and segmental angles significantly decreased in all sitting positions, especially in unsupported postures, while SS decreased and PT increased in tandem with the loss of LL [10]. Another prospective comparative study of 58 healthy individuals presenting with recent-onset lower back pain reported a mean LL reduction of 24.6° and a 50% increase in PT [7]. Additionally, a third study examining 50 healthy adults found an average decrease in LL and SS of approximately 50% and an increase in PT by nearly 285% in the sitting position compared to standing [9]. However, one of the most important findings of this study, which was not detected in the previous literature, was the significant change in PI between these positions, with higher values observed in the sitting position compared to standing. Despite PI being traditionally considered a fixed anatomical parameter [29], we observed a mean ΔPI of −4.52° (*p* < 0.001), with PI increasing by 8.8% in sitting compared to standing. This motion is likely mediated through the sacroiliac joint (SIJ) [16,30,31]. This finding suggests that PI may not be as static as previously thought and can be influenced by postural changes, demonstrating SIJ-mediated postural adaptation in ASD patients [32,33,34]. This change calls for attention to preoperative SIJ mobility assessments in ASD surgery to avoid spinopelvic fixation failure [34,35,36,37] and suggests intraoperative PI adjustments based on sitting posture.

The analysis showed that age, BMI, and gender were independently associated with changes in spinopelvic parameters. Age significantly diminished spinopelvic adaptability, with older adults exhibiting compensatory stiffness and limited mobility [38,39]. Older age was related to increased ΔPT and ΔPI but decreased ΔLL and ΔSS. Patients ≥ 70 years had 47% smaller ΔLL than those < 60 and 32% smaller ΔSS, consistent with age-related spinal degeneration. These findings are consistent with the past literature, which has documented age-related changes in spinal alignment [40,41]. The age-related changes observed in this study may be attributed to different factors, including degenerative spinal changes, muscle weakness, and postural control [14,33,34]. In younger patients with spinal fusion, there is an increased reliance on hip motion, resulting in greater mechanical loads on the hips compared to elderly patients, who have reduced mobility and increased spine stiffness [42,43]. This suggests that older adults may have a similar limited capacity to accommodate sagittal imbalance through these compensatory mechanisms. The BMI effect was also significant, as higher BMI was associated with reduced postural adaptability, particularly in PT. Obese patients (BMI ≥ 30) showed 23% smaller ΔPT than normal-weight individuals. While PT in the standing position may not differ significantly in different BMI groups, obese individuals tend to exhibit more pelvic retroversion and recruit greater posterior PT when sitting, likely to compensate for soft-tissue impingement and altered trunk mass distribution [44,45]. The observed decreases in ΔPT and ΔPI may be linked to reduced spinopelvic motion, possibly due to restrictions from increased abdominal adiposity [46]. The observed reductions in both ΔPT and ΔPI in obese individuals may reflect a mechanical limitation in sacropelvic motion due to increased visceral and subcutaneous fat mass, consistent with prior biomechanical and imaging studies [44,47]. Gender differences were also significant, with females showing 14% greater ΔPT than males but 11% smaller ΔLL. These findings match previous studies that reported gender-related differences in lumbar alignment, with women generally having greater lumbar flexibility [9,48,49], which may explain their reliance on pelvic retroversion for balance [49,50]. This tendency may reflect inherent differences in pelvic morphology and spinal compliance, as well as hormonal and connective tissue influences [48,49,51]. Taken together, surgical planning in elderly patients should prioritize preserving native alignment to minimize adjacent-segment stress while also accounting for restricted pelvic mobility in obese patients to avoid overcorrecting lumbar lordosis; additionally, gender-specific alignment targets may optimize outcomes, particularly in females’ hypermobile lumbar regions.

It is important to acknowledge some limitations of this study. First, the study group consisted of patients with HOA, which may have influenced spinopelvic alignment through altered compensatory mechanisms [19]. However, prior studies indicate that the degree of pelvic retroversion involved in transitioning from standing to sitting—functionally synonymous with hip flexion and extension—remains within a preserved range of motion even in individuals with moderate to severe hip arthritis [21,30,52]. Therefore, while HOA may contribute to some variability, the motion required for sitting radiographs likely remained physiologically feasible across the cohort [14,53]. Second, the absence of Patient-Reported Outcome Measures (PROMS) limits the ability to correlate radiographic changes with pain or functional outcomes. Additionally, given the study group’s age, BMI, and surgical status, chronic lumbar back pain could have contributed to compensatory spinopelvic adaptations [19,20]. However, as an HOA, chronic lumbar pain is common in older adults [14,54]; we believe this cohort closely reflected the typical phenotype of patients undergoing ASD surgery.

## 5. Conclusions

This study represents the largest series of patients radiographically measured in both sitting and standing positions, providing a comprehensive comparison of significant changes in spinopelvic parameters between these two postures. The findings indicated an increase in PI and PT, as well as a decrease in SS and LL in the sitting position. Additionally, age, BMI, and gender were found to be independent factors influencing the extent of spinopelvic motion, highlighting the need to incorporate these variables when reconstructing spinal alignment. Notably, the significant change observed in PI between sitting and standing raises important clinical considerations regarding SIJ mobility and the implications for SIJ fusion. Given these results, key recommendations include the preoperative assessment of SIJ motion to inform fusion decisions and the establishment of demographic-specific alignment targets based on age, BMI, and gender.

## Figures and Tables

**Table 1:** **Demographic and radiological summary.** Summarizes demographics and radiographic data of different spinopelvic parameters in sitting and standing positions. All parameters with changing from sitting to standing position had a significant difference (*p* < 0.001).

Variable	Overall ^1^
Number of Patients	1447
Age (years)	63.59 (10.85)
BMI (kg/m^2^)	28.81 (5.70)
Gender (%) Female	795 (54.9)
Male	652 (45.1)
Standing PI (°)	51.67 (10.75)
Standing PT (°)	14.21 (8.99)
Standing SS	37.46 (8.59)
Standing LL (°)	48.49 (12.21)
Standing PI-LL (°)	3.18 (12.63)
Sitting PI (°)	56.19 (10.75)
Sitting PT (°)	33.41 (11.41)
Sitting SS (°)	22.78 (8.79)
Sitting LL (°)	30.05 (11.92)
Sitting PI-LL (°)	26.14 (13.97)
ΔPI (°)	−4.52 (5.68)
ΔPT (°)	−19.20 (11.00)
ΔSS (°)	14.67 (10.18)
ΔLL (°)	18.44 (13.29)

^1^ Continuous variables were summarized using means (standard deviations), while categorical variables were presented as frequencies (percentages). PI = pelvic incidence; PT = pelvic tilt; SS = sacral slope; LL = lumbar lordosis; Δ = change from sitting to standing; PT–LL = pelvic tilt minus lumbar lordosis; PI–LL = pelvic incidence minus lumbar lordosis.

**Table 2:** **Age subgroup analysis.** Stratified data across three age subgroups: Group 1 (under 60 years), Group 2 (60–69 years), and Group 3 (70 years or older).

Variable	Group 1 ^1^	Group 2 ^1^	Group 3 ^1^	*p*-Value
Number of Patients	490	531	426	
Age (years)	51.88 (7.11)	64.65 (2.84)	75.72 (5.05)	**<0.001**
BMI (kg/m^2^)	29.94 (6.13)	28.71 (5.63)	27.65 (5.00)	**<0.001**
Gender (%) Female	260 (53.1)	280 (52.7)	255 (59.9)	0.052
Male	230 (46.9)	251 (47.3)	171 (40.1)	
ΔPI (°)	−4.90 [−8.17, −1.50]	−3.90 [−7.90, −0.70]	−4.20 [−7.27, −0.60]	**0.027**
ΔPT (°)	−23.00 [−30.87, −14.03]	−18.10 [−24.85, −11.10]	−15.20 [−22.08, −8.60]	**<0.001**
ΔSS (°)	17.15 [9.90, 25.20]	13.70 [7.65, 20.75]	10.45 [5.22, 16.70]	**<0.001**
ΔLL (°)	24.95 [14.18, 33.18]	17.90 [9.35, 26.00]	12.90 [4.60, 20.20]	**<0.001**

^1^ Normally distributed continuous variables were reported as mean (SD), while non-normal data were presented as median [IQR]. Percentages were used for categorical variables, with *p*-values indicating significant subgroup differences. PI = pelvic incidence; PT = pelvic tilt; SS = sacral slope; LL = lumbar lordosis; Δ = change from sitting to standing.

**Table 3:** **BMI subgroup analysis.** Stratified data across three BMI subgroups: Group 1 (normal weight), Group 2 (overweight), and Group 3 (obese). This table shows mean values with SDs, median values with IQRs for continuous variables, and percentages for categorical variables, accompanied by *p*-values to indicate the statistically significant subgroup differences.

Variable	Group 1 ^1^	Group 2 ^1^	Group 3 ^1^	*p*-Value
Number of Patients	397	498	546	
Age (years)	64.96 (10.75)	64.89 (11.53)	61.51 (9.88)	**<0.001**
BMI (kg/m^2^)	22.47 (1.90)	27.48 (1.42)	34.63 (4.07)	**<0.001**
Gender (%) Female	295 (74.3)	235 (47.2)	263 (48.2)	**<0.001**
Male	102 (25.7)	263 (52.8)	283 (51.8)	
ΔPI (°)	−3.90 [−7.40, −1.00]	−3.85 [−6.90, −0.52]	−5.05 [−9.10, −1.50]	**<0.001**
ΔPT (°)	−16.40 [−24.80, −9.70]	−18.10 [−25.15, −10.83]	−20.10 [−28.58, −12.60]	**<0.001**
ΔSS (°)	11.80 [6.40, 19.80]	13.95 [7.32, 21.78]	14.65 [8.03, 22.90]	**0.003**
ΔLL (°)	16.70 [8.40, 25.60]	19.00 [9.03, 27.98]	18.50 [8.90, 28.48]	0.065

^1^ Presenting mean (SD), median [IQR] for continuous variables, and percentages for categorical variables, with *p*-values highlighting significant subgroup differences. PI = pelvic incidence; PT = pelvic tilt; SS = sacral slope; LL = lumbar lordosis; Δ = change from sitting to standing.

**Table 4:** **Gender-based analysis.** Stratified by gender subgroups (females and males).

Variable	Female ^1^	Male ^1^	*p*-Value
Number of Patients	795	652	
Age (years)	64.26 (10.69)	62.77 (11.00)	**0.009**
BMI (kg/m^2^)	27.95 (5.99)	29.86 (5.14)	**<0.001**
ΔPI (°)	−4.40 [−7.90, −1.20]	−4.10 [−7.70, −0.70]	0.178
ΔPT (°)	−17.40 [−25.00, −10.60]	−19.80 [−28.22, −11.78]	**0.001**
ΔSS (°)	12.60 [6.60, 20.40]	15.50 [8.10, 23.20]	**<0.001**
ΔLL (°)	17.30 [8.40, 25.75]	19.40 [9.90, 29.20]	**0.001**

^1^ Presenting mean (SD), median [IQR] for continuous variables, and percentages for categorical variables, accompanied by *p*-values to demonstrate the statistically significant subgroup differences. PI = pelvic incidence; PT = pelvic tilt; SS = sacral slope; LL = lumbar lordosis; Δ = change from sitting to standing.

## Data Availability

The data presented in this study are available on request from the corresponding author due to institutional policies and patient confidentiality regulations. Data sharing is restricted to protect sensitive patient information in compliance with ethical guidelines and IRB-approved protocols.

## Abbreviations

The following abbreviations are used in this manuscript:

ASD	Adult Spinal Deformity
ASA	American Society of Anesthesiologists
BMI	Body Mass Index
CCI	Charlson Comorbidity Index
HOA	Hip Osteoarthritis
HR	Hip Replacement
HSS	Hospital for Special Surgery
IQR	Interquartile Range
IRB	Institutional Review Board
LL	Lumbar Lordosis
NIH	National Institute of Health
OA	Osteoarthritis
PI	Pelvic Incidence
PI-LL	Pelvic Incidence minus Lumbar Lordosis
PT	Pelvic Tilt
PROMS	Patient-Reported Outcome Measures
REDCap	Research Electronic Data Capture
SD	Standard Deviation
SIJ	Sacroiliac Joint
SS	Sacral Slope
XR	X-ray
